# Comparative assessment of Mini-FLOTAC, McMaster and semi-quantitative flotation for helminth egg examination in camel faeces

**DOI:** 10.1186/s13071-024-06637-3

**Published:** 2025-01-12

**Authors:** Khalid M. Mohammedsalih, Salma A. Hassan, Fathel-Rahman Juma, Shamsaldeen I. Saeed, Ahmed Bashar, Georg von Samson-Himmelstjerna, Jürgen Krücken

**Affiliations:** 1 Institute for Parasitology and Tropical Veterinary Medicine, Freie Universität Berlin, Robert-von-Ostertag-Str. 7, 14163 Berlin, Germany; 2 Veterinary Centre for Resistance Research, Freie Universität Berlin, 14163 Berlin, Germany; 3 Central Research Laboratory of Darfur Universities, Mousseh District, 63311 Nyala, Sudan; 4 Faculty of Veterinary Science, University of Nyala, Mousseh District, 63311 Nyala, Sudan

**Keywords:** Helminth infections, Faecal egg quantification, Test accuracy, Dromedary

## Abstract

**Background:**

Faecal egg counts (FECs) are essential for diagnosing helminth infections and guiding treatment decisions. For camels, no evaluations of coproscopic methods regarding precision, sensitivity and correlation between individual and pooled faecal samples are currently available.

**Methods:**

Here, 410 camel faecal samples were collected in 2022 from South Darfur State, Sudan, and analysed to compare the semi-quantitative flotation, McMaster and Mini-FLOTAC methods in terms of precision, sensitivity, inter-rater reliability and helminth egg count correlations, as well as the effects of pooling samples. Six samples were used to assess precision for McMaster and Mini-FLOTAC, while the remaining 404 samples were evaluated for sensitivity, inter-rater reliability and egg count correlations. Of these, 80 samples were used in pooling experiments.

**Results:**

Six analyses of each sample (*n* = 6) using the McMaster and Mini-FLOTAC methods revealed no significant difference in the coefficient of variation between the two. For strongyle eggs, 48.8%, 52.7% and 68.6% were positive for McMaster, semi-quantitative flotation and Mini-FLOTAC, respectively. The sensitivity of the three methods showed only minimal improvement when three egg counts were performed on the same sample. McMaster and Mini-FLOTAC had similar sensitivity for *Strongyloides* spp. (3.5% frequency), while it was lower for semi-quantitative flotation at 2.5%. Mini-FLOTAC was more sensitive for *Moniezia* spp., detecting 7.7% of positives compared with 4.5% for semi-quantitative flotation and 2.2% for McMaster. For *Trichuris* spp., frequencies were 0.3% with Mini-FLOTAC, 0.7% with McMaster and 1.7% with semi-quantitative flotation. Mini-FLOTAC also detected higher strongyle eggs per gram (EPG) of faeces (mean 537.4) compared with McMaster (330.1). More samples exceeded treatment thresholds with Mini-FLOTAC, with 28.5% of animals having EPG ≥ 200 compared with 19.3% for McMaster, while 19.1% showed EPG ≥ 500 with Mini-FLOTAC compared with 12.1% with McMaster. There was no significant correlation between individual and pooled strongyle FECs, as indicated by Pearson correlation coefficients of *r* ≥ 0.368 (*P* ≥ 0.113) and Spearman correlation.

**Conclusions:**

Mini-FLOTAC outperformed semi-quantitative flotation and McMaster in diagnosing helminth infections in camels, offering greater sensitivity and detecting higher EPGs, particularly for strongyles, *Strongyloides* spp. and *Moniezia* spp. Thus, treatment decisions based on Mini-FLOTAC EPGs will lead to more treatments.

**Graphical Abstract:**

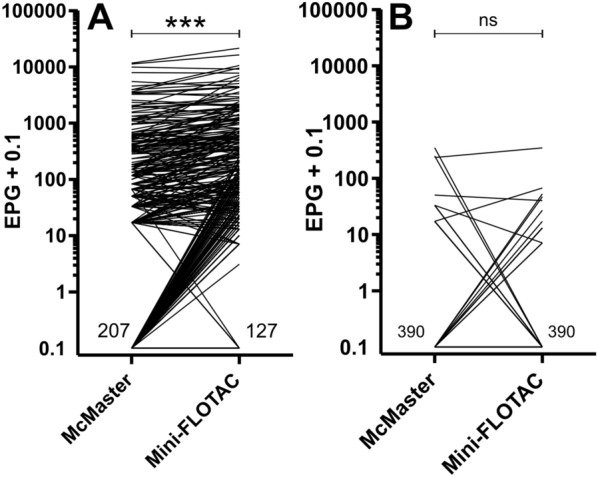

**Supplementary Information:**

The online version contains supplementary material available at 10.1186/s13071-024-06637-3.

## Background

Helminths such as nematodes, cestodes and trematodes represent a significant challenge, causing some of the most prevalent, diverse and detrimental diseases affecting both humans and animals and resulting in substantial socio-economic and health impacts, particularly in animals in tropical regions, including Sudan [1, 2].

To improve animal health and optimise anthelmintic use, helminth infections should be monitored regularly through screening strategies. Faecal egg counting remains a fundamental diagnostic method in veterinary medicine for clinical diagnosis, assessing treatment efficacy, guiding targeted treatment and detecting co-infections [3]. Diagnosis mainly relies on microscopy of faecal or urine samples, using wet mounts or concentration techniques. Concentration methods include flotation and sedimentation, with flotation subdivided into test-tube and coverslip method (semi-quantitative) or counting chamber approaches (quantitative). The McMaster method, developed in the 1930s [4], remains a standard and has inspired modifications including FECPAK, FLOTAC and Mini-FLOTAC [5, 6]. The rapid emergence of anthelmintic resistance in ruminants [7] and horses [8] highlights the need for surveillance-based control strategies to minimise routine anthelmintic use and limit resistance.

Effective helminth control in grazing animals such as ruminants and camelids relies on regular monitoring through faecal egg counts (FECs) and assessing treatment efficacy using faecal egg count reduction tests (FECRTs). However, the sub-clinical nature of infections and limited awareness of anthelmintic resistance often lead to the misconception that monitoring is too time-consuming and costly, resulting in treatments being given without diagnosis [9]. Composite sampling, where faecal samples from multiple animals are pooled for analysis, offers a promising alternative, less costly solution. While studies have examined its correlation with individual sampling for infection burden and treatment efficacy [9–11], data on its use in several livestock species, including camels, remain limited or missing.

The dromedary camel (*Camelus dromedarius*), known as the “animal of the future”, is highly resilient and adaptable to arid regions where crop and livestock production struggle owing to erratic rainfall and climate change [12]. Camels support food security by providing dairy, meat, wool, hair, hides and transport [13]. Despite their resilience, camels are vulnerable to pathogens, especially gastrointestinal helminths, including gastrointestinal nematodes (GINs) [14]. Some GINs are camel-specific, such as *Physocephalus dromedarii* and *Nematodirus dromedarii*, while others, including *Haemonchus longistipes* and *Trichostrongylus vitrinus*, are shared with sheep and goats [15, 16]. Among these, *Haemonchus* spp. are particularly pathogenic, causing anaemia, hypoproteinaemia, weight loss and death in young camels owing to their blood-feeding behaviour [17, 18].

Routine assessment of anthelmintic treatment efficacy by means of FECRTs is crucial. However, interpreting these tests requires distinguishing true egg count reductions from random data variations, with the choice of egg counting technique significantly influencing test outcomes [19]. In particular, Mini-FLOTAC has been introduced as a viable alternative to the traditional McMaster technique, offering simultaneous identification of helminth eggs with higher sensitivity, accuracy and precision [20, 21]. While Mini-FLOTAC has been implemented into routine parasitological diagnosis across various animal species since its introduction in 2013 [5], it has so far not been applied in epidemiological studies involving camels. Furthermore, no consensus has been reached on the best diagnostic protocol for helminth infections in camels. This study aimed to compare the semi-quantitative flotation method (test tube and coverslip) with the quantitative methods, McMaster and Mini-FLOTAC, in terms of precision, sensitivity, inter-rater reliability, correlation of helminth egg counts and pooling. The goal is to enhance the selection of optimal diagnostic tools for camel faecal samples, particularly for nematodes.

## Methods

### Study location and design

**Fig. 1:**
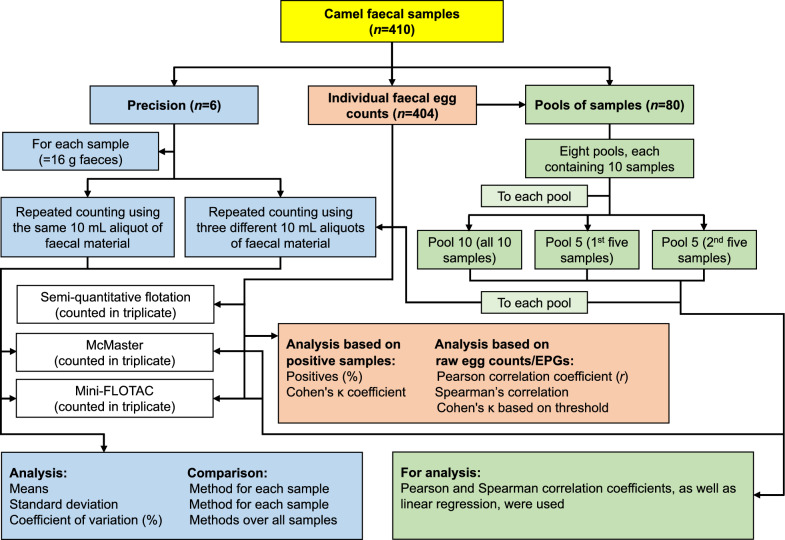
Diagram visualisation of the study design. The diagram includes three main aspects: (i) Precision evaluation of the two quantitative methods, viz. McMaster and Mini-FLOTAC. This experiment involved six samples with different EPG of faeces. For each method, the faecal material extracted from each sample was divided into four 10-mL aliquots and placed in four 15-mL test tubes. Each sample was counted six times using both methods to estimate precision. Egg counting was performed three times with the first 10-mL aliquot (tube 1) and three times with the remaining three aliquots. Mean and SD values were compared for each method across all samples, and the CV (%) was compared between the methods. (ii) Samples (*n* = 404) were analysed once with each of the three methods, viz. semi-quantitative flotation, McMaster and Mini-FLOTAC, in triplicate using three faecal material aliquots from three different 15-mL test tubes. (iii) Eighty faecal samples from study (ii) were utilised to compare individual and pooled faecal samples. Pools including ten samples each were prepared (*n* = 8 pools), and subsequently, each pool was subdivided into two different pool sizes, each containing five samples. Each of the three pools (the original pool of ten and the two sub-sized pools of five) was analysed using the McMaster and Mini-FLOTAC methods to assess gastrointestinal strongyle intensity. On the basis of the presence or absence of helminth eggs, the percentage of positive samples and inter-rater agreement, using Cohen’s *κ* values, were compared. Utilising quantitative data on EPG or raw egg counts, Pearson and Spearman correlations between methods were calculated. Samples were then assigned to categories based on thresholds derived from the quantitative methods, that is, the McMaster and Mini-FLOTAC. The assignment of samples to these categories was analysed using Cohen’s *κ* statistics. *CV* coefficient of variation, *EPG* eggs per gram of faeces, *SD* standard deviation

This study was conducted on camels (*C. dromedarius*) in South Darfur State, southwest Sudan, during the rainy season from August to October 2022 (a savannah climate), with the camel population estimated at around 89,895 [22]. A total of 410 faecal samples were obtained by convenience sampling from camels at the livestock market of Nyala (12.05° N 24.88° E), the capital of South Darfur State [23]. These samples were used to: (i) validate three diagnostic methods (semi-quantitative flotation, McMaster and Mini-FLOTAC) by analysing their sensitivity for detecting helminth eggs and (ii) assess the precision of these methods. Additionally, the samples were used to evaluate the effects of different pooling strategies (Fig. 1). The study included male and female camels aged between 6 months and over 12 years (based on dentition [24]), comprising indigenous breeds raised under a free-range husbandry system. Faecal samples were directly collected from the rectum using disposable gloves, placed in labelled plastic bags and then transferred in a cooling bag to the Central Research Laboratory of Darfur Universities, at the University of Nyala, Nyala, Sudan. Upon arrival, the samples were stored in a refrigerator (4 °C) for a maximum of 24 h before egg counting. Typically, 50 samples were collected during each of the nine visits to the livestock market, and all were examined on the same day using the three methods. For the precision study, all replicates of the same sample were investigated on the same day.

### Coproscopical techniques

All collected faecal samples (*n* = 410) were processed and analysed using the semi-quantitative flotation, McMaster and Mini-FLOTAC methods in triplicates. All faecal samples from each animal were initially homogenised using a pestle and mortar before being subjected to coproscopical diagnosis.

#### Semi-quantitative flotation method

This semi-quantitative method detects nematode and cestode eggs. Following Gibbons et al. [25], 6 g of homogenised faeces (twice the standard weight) was weighed using a 0.001 g sensitive balance (Shimadzu BL220H Top Loading Balance, Shimadzu Corporation, Kyoto, Japan). The faeces were mixed with 100 mL of saturated sodium chloride solution (relative density 1.20 [26]) and filtered through a 0.3-mm mesh strainer into a 400-mL beaker. The suspension was distributed into three 15-mL test tubes for triplicate counting, topped with a convex meniscus, and covered with a coverslip for 20 min. The coverslip was then transferred to a slide and examined under a light microscope (Olympus CX23, Olympus Corporation, Shinjuku, Japan). Helminth eggs were identified and counted using standard keys [27]. Egg count results were categorised as: negative (no eggs), + (1–10 eggs), ++ (11–40 eggs), +++ (41–200 eggs), and ++++ (> 200 eggs). The operating steps for this method are summarised in Additional file 1: Fig. S1A.

#### McMaster quantitative flotation

This quantitative method was employed as a reference technique owing to its historical and frequent use in parasitology laboratories. Following Gibbons et al. [25] and MAFF [28], 6 g of homogenised faeces (twice the standard weight) was weighed and mixed with 84 mL of saturated sodium chloride solution (relative density 1.2). The mixture was filtered through a 0.3-mm mesh strainer into a 400-mL beaker, then divided into three or four 10-mL aliquots as required and placed in 15-mL test tubes for repeated egg counting. Each aliquot was used to fill two counting chambers (0.15 mL each), and eggs were allowed to float for 10 min and then counted. The eggs per gram (EPG) values were calculated by multiplying the observed egg count by 50. The operating steps for this method are summarised in Additional file 1: Fig. S1B.

#### Mini-FLOTAC

Mini-FLOTAC, a quantitative FEC method proposed as a replacement for McMaster, was performed according to the manufacturer’s guidelines for small ruminants. Ten grams of homogenised faeces (twice the standard weight) was mixed with 90 mL of saturated sodium chloride solution (specific gravity 1.20) and filtered through a 0.3-mm mesh strainer into a 400-mL beaker. The faecal material was divided into three or four 10-mL aliquots and transferred to 15-mL test tubes for repeated egg counting. From each aliquot, 2 mL of suspension was added to two Mini-FLOTAC chambers and left to float for 10 min [21]. Helminth eggs were identified and counted under a light microscope [27], and the EPG was calculated by multiplying the egg count by 5. The operating steps for the Mini-FLOTAC method are summarised in Additional file 1: Fig. S1C.

### Precision of the methods

In this experiment, a precision analysis was conducted for the quantitative FEC methods, that is, McMaster and Mini-FLOTAC. Six camel faecal samples were collected to assess the precision of these two methods. The analysis involved subsampling from thoroughly mixed faecal samples. For each sample, 16 g of faeces was weighed and divided between the two methods (6 g for McMaster and 10 g for Mini-FLOTAC). Each sample was prepared for egg counting using the above-described procedure for either the McMaster or Mini-FLOTAC method. For each method, 40 mL of filtered faecal material was divided into four 10-mL aliquots in 15-mL test tubes. Each sample was counted six times with each method to estimate precision. Egg counting was repeated three times using the first 10-mL aliquot (tube 1) and three times using the remaining three aliquots (Fig. 1).

### Sensitivity of the semi-quantitative flotation, McMaster and Mini-FLOTAC methods on individual faecal egg counts

The sensitivity of the three methods was assessed using a total of 404 samples, each subjected to individual FECs. Each sample was examined by all three methods in triplicate, using three faecal material aliquots from three different 15-mL test tubes (Fig. 1).

### Pools of samples

To assess the abundance of strongyle egg shedding in camels, both individual and pooled faecal samples were examined. In this experiment, faecal samples were collected from 80 individual camels, all contributing to the total faecal samples (*n* = 404) used to evaluate the sensitivity of the three methods (Fig. 1). Initially, each sample underwent individual examination for the presence of strongyles (with Mini-FLOTAC and McMaster in triplicate). Subsequently, samples were grouped based on FECs obtained using the Mini-FLOTAC method into negative, less than 200 EPG, 200–500 EPG, 500–1000 EPG and more than 1000 EPG. The samples were then distributed into eight groups, each containing ten samples, ensuring representation from each of the initial five EPG categories. For the preparation of pools, 30 g of faeces was taken from each sample. Each of the eight pool groups was then combined into one composite sample, comprising ten individual faecal samples (10 g from each sample), and two composite samples, each consisting of five individual faecal samples (10 g from each sample for each of the two pools). The composite faeces of each pool were homogenised using a pestle and mortar and analysed with each of the two quantitative coproscopic methods, viz. McMaster and Mini-FLOTAC, in triplicate. This analysis utilised three faecal material aliquots (10 mL each) from three different 15-mL test tubes (Fig. 1).

### Statistical analyses

The raw egg counts and EPG data were collected and stored in Microsoft Excel. The data were statistically analysed using R software version 4.3.2 and the graphical user interface RStudio version 2023.12.1. GraphPad Prism version 8.0.2 for Windows was also used for data analysis and plotting of the data. A value of *P* < 0.05 was considered significant for all statistical tests. The performance of the three diagnostic methods for detecting helminth infections in camel faeces was assessed using combined results as the “gold standard” [29]. Average values from three replicates per sample and method were computed in Excel, adjusted (0.1–0.9 set to 1.0), converted to integers and transformed into binomial variables: 1 (positive, > 0 egg counts) and 0 (negative). Helminth egg burden was classified as low (< 200 EPG), mild (200–500 EPG), moderate (500–1000 EPG) or high (> 1000 EPG) [30, 31]. Precision was analysed by comparing raw egg counts and EPG using analysis of variance (ANOVA) with Tukey’s test in GraphPad Prism. Bartlett’s test in R was applied to assess variance differences among methods for the same sample. Coefficients of variation (CVs) were calculated for each method/sample and compared across methods using Friedman’s test and Tukey’s post hoc test. Positive sample proportions were calculated with Wilson score intervals (epitools package version 0.5–10.1 in R). Pairwise comparisons were performed using mid-p exact tests, with *P* values adjusted for multiple testing using the p.adjust() function in R, applying the “Holm” method. Mid-p exact tests were also used to evaluated whether multiple tests identified more positive samples than single tests. Cohen’s *κ* statistics were used to assess the agreement between the semi-quantitative flotation, McMaster and Mini-FLOTAC methods for positive/negative identification (CohenKappa() function from DescTools package version 0.99.48 in R), with agreement levels classified as no (≤ 0), poor (0.01–0.20), fair (0.21–0.40), moderate (0.41–0.60), substantial (0.61–0.80) or almost perfect (≥ 0.81) agreement [32]. For pooled samples, mean EPGs from individual samples were compared with pooled EPGs using Pearson and Spearman correlations and linear regression in GraphPad Prism. This was performed independently for the different methods (McMaster and Mini-FLOTAC) and pool sizes (five or ten samples).

## Results

### Comparison of precision between the McMaster and Mini-FLOTAC methods

**Fig. 2:**
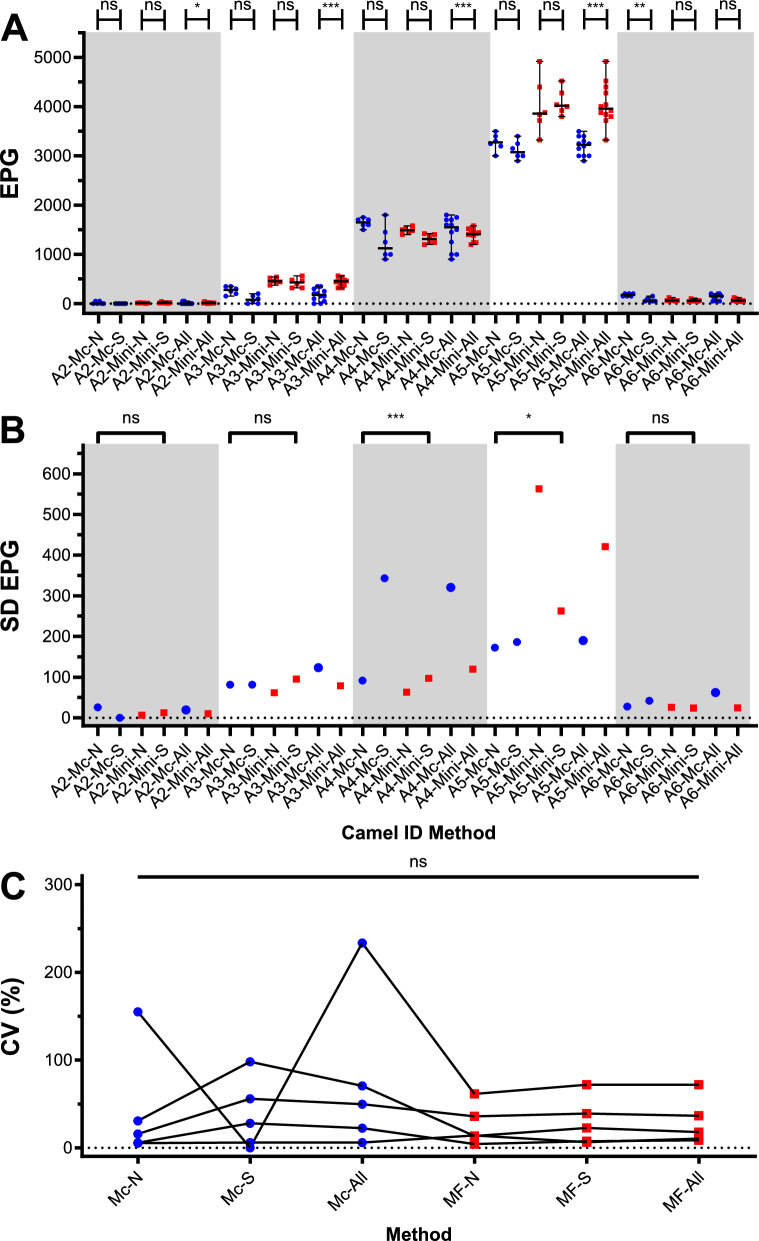
Comparison of precision between McMaster and Mini-FLOTAC methods. This experiment used six samples with varying strongyle EPG of faeces. For each method, viz. McMaster and Mini-FLOTAC, the extracted faecal material from each sample was divided into four 10-mL aliquots and placed in four 15-mL test tubes. Each sample was counted six times with each method to estimate precision. Egg counting was performed three times using the first 10-mL aliquot (tube 1) and three times using the remaining three aliquots. Data from animal 1 were excluded as no helminth eggs were detected. **A** EPG of six replicates (3 + 3) were compared for each sample (*n* = 5) using one-way ANOVA and Tukey’s multi-comparison test. **B** SD for six replicates was compared using Bartlett’s test. **C** CV (%) was calculated for each sample and method; no significant differences were found (*P* < 0.05, Friedman test). McMaster results are shown as blue circles, and Mini-FLOTAC as red squares. A-(2, …)-Mc-N, three counts using McMaster with different aliquots; A-(2, …)-Mc-S, three counts using McMaster with the same aliquot; A-(2, …)-Mc-All, EPG combined from six McMaster replicates (3 + 3); A-(2, …)-Mini-N, three counts using Mini-FLOTAC with different aliquots; A-(2, …)-Mini-S, three counts using Mini-FLOTAC with the same aliquot; A-(2, …)-Mini-All, EPG combined from six Mini-FLOTAC replicates (3 + 3), *ANOVA* analysis of variance, *CV* coefficient of variation, *EPG* eggs per gram of faeces, *SD* standard deviation; *ns*, not significant; ***, **, *, *P* < 0.001, 0.01, 0.05, respectively

Data for animal 1 were excluded from the statistical analysis since all examinations were negative for helminth eggs. Repeated egg counts using faecal material from the same 10-mL aliquot or from three different 10-mL aliquots showed high similarity among the results for both approaches. The Kruskal–Wallis analysis with Dunn’s post hoc tests comparing results between data obtained using counts from the same and different tubes and for all replicates between Mini-FLOTAC and McMaster revealed only a significant difference (*P* < 0.01) for sample 6 for the McMaster technique between samples drawn from the same or different aliquots (Fig. 2A). However, there were four significant differences between Mini-FLOTAC and McMaster (samples 2, 3, 4 and 5) (Fig. 2A). For this analysis, all six egg counts (three from tube 1 and one from each of the three different remaining 10-mL aliquots) were included. For three samples (nos. 2, 3 and 5), the mean and median of the EPG were higher for the Mini-FLOTAC than for McMaster method (Fig. 2). For sample 4, both the mean and median values were slightly higher when using the McMaster method. Generally, the SD between the two methods was quite similar (Fig. 2B), but there were notable outliers that contributed to significant differences (*P* < 0.05) in samples 4 and 5 (Fig. 2B). For sample 2, however, the SD could not be calculated for the McMaster method because all readings from the same 10-mL aliquots were zero. The coefficient of variation values were calculated to compare the relative variation dependent on the number of eggs counted. Values ranged from 0% to 233.5% for McMaster and from 4.2% to 71.9% for Mini-FLOTAC (Fig. 2C). Kruskal–Wallis followed by Dunn’s multiple comparison test did not reveal any significant differences (*P* < 0.05) regarding the size of the CV between the two methods (Fig. 2C).

### Number of positive samples and faecal egg counts for different helminth groups

The faecal samples examined in this study, including the precision samples, were of normal consistency, firm and dry. The sensitivity of the three evaluated methods for the detection of various helminth genera/groups in camel faeces (*n* = 404) is presented in Table 1. Additionally, this table includes the median and interquartile range (IQR) of raw egg counts or EPGs obtained using the semi-quantitative flotation, McMaster and Mini-FLOTAC methods, respectively. These methods differentiated three GIN genera/groups, including strongyles, *Strongyloides* spp., and *Trichuris* spp., as well as *Moniezia* spp. cestodes. There was no evidence for the presence of *Nematodirus* spp. and *Capillaria* spp. On the basis of triplicate readings, the overall average frequency of helminths was 50.3%, 55.2% and 71.0% for the McMaster, semi-quantitative flotation and Mini-FLOTAC methods, respectively (Table 1). The frequency of samples positive for any helminth was significantly higher for Mini-FLOTAC than for McMaster or semi-quantitative flotation (*P* < 0.001, mid-p exact test) while the difference between McMaster and semi-quantitative flotation was not significant (*P* = 0.160). For strongyle eggs, the same significant differences were observed. For *Strongyloides* spp. and *Trichuris* spp., there were no significant differences between the three methods after adjustment of the *P* values for multiple testing. In contrast, Mini-FLOTAC was significantly more sensitive for the detection of *Moniezia* spp. than McMaster (*P* < 0.001), while differences between semi-quantitative flotation and Mini-FLOTAC or McMaster were not significant. Samples were assigned to different categories of infection using either Mini-FLOTAC or McMaster (Table 1). For all four categories, the number of samples was higher for Mini-FLOTAC than for McMaster.

**Table 1:** Numbers, relative frequencies of samples positive for the respective helminth species/group and eggs per gram faeces in camel faecal samples (*n* = 404) analysed using the semi-quantitative flotation, McMaster and Mini-FLOTAC methods

Mini-FLOTACTest procedure	All helminths	Gastrointestinal nematodes				*Moniezia* spp.	Gastrointestinal nematode burden			
		All	Strongyles	*Strongyloides* spp.	*Trichuris* spp.		Low^b^	Mild^b^	Moderate^b^	High^b^
*Mini-FLOTAC*										
Number positive	287	279	277	14	1	31	164	38	29	48
Frequency (%)	71.0	69.1	68.6	3.5	0.3	7.7	40.6	9.4	7.2	11.9
95% CI (%)^a^	66.4–75.3	64.4–73.4	63.9–72.9	2.1–5.7	0.04–1.4	5.5–10.7	35.9–45.5	6.9–12.7	5.0–10.1	9.1–15.4
Median EPG	40.0	33.0	33.0	0	0	0	33.0	283.5	613.0	2040.0
IQR EPG	0–296.8	0–271.5	0–271.5	0	0	0	17.0–73.0	238.3–368.5	570.0–717.0	1360.0–4404.0
Range EPG	0–21587.0	0–21587.0	0–21587.0	0–347.0	0–7.0	0–1893.0	3.0–173.0	200.0–493.0	507.0–947.0	1013.0–21,587.0
*McMaster*										
Number positive	203	202	197	14	3	9	124	28	21	29
Frequency (%)	50.3	50.0	48.8	3.5	0.7	2.2	30.7	6.9	5.2	7.2
95% CI (%)^a^	45.4–55.1	45.2–54.9	43.9–53.6	2.1–5.7	0.3–2.2	1.2–4.2	26.4–35.4	4.8–9.8	3.4–7.8	5.0–10.1
Median EPG	17.0	8.5	0	0	0	0	50.0	350.0	633.0	2347.0
IQR EPG	0–117.0	0–117.0	0–117.0	0	0	0	17.0–100.0	317.0–400.0	567.0–767.0	1442.0–4059.0
Range EPG	0–11650.0	0–11650.0	0–11650.0	0–350.0	0–17.0	0–733.0	17.0–183.0	200.0–483.0	500.0–983.0	1100.0–11,650.0
*Semi-quantitative flotation*										
Number positive	223	217	213	10	7	18	n.a	n.a	n.a	n.a
Frequency (%)	55.2	53.7	52.7	2.5	1.7	4.5	n.a	n.a	n.a	n.a
95% CI (%)^a^	50.3–60.0	48.8–58.5	47.9–57.5	1.4–5.5	0.8–3.5	2.8–6.9	n.a	n.a	n.a	n.a
Median raw egg counts	1.0	1.0	1.0	0	0	0	n.a	n.a	n.a	n.a
IQR raw eggs	0–2.0	0–2.0	0–2.0	0	0	0	n.a	n.a	n.a	n.a
Range raw eggs	0–82.0	0–82.0	0–82.0	0–1.0	0–1.0	0–11.0	n.a	n.a	n.a	n.a

^a^95% confidence intervals calculated as Wilson score intervals

^b^Gastrointestinal nematode burdens using McMaster and Mini-FLOTAC methods; low infection: < 200 eggs/gram faeces (EGP), mild infection: 200–500 EPG, moderate infection: 500–1000 EPG and high infection > 1000 EPG

*EPG* eggs per gram faeces, *IQR* interquartile range

### Assessment of repeated egg counts using semi-quantitative flotation, McMaster and Mini-FLOTAC methods

The potential increase in sensitivity that may have been obtained by testing each sample three times was investigated for all three methods, focussing on strongyle and *Moniezia* spp. eggs (Table 2). For this purpose, mid-p exact tests were conducted, comparing the percentage of positive samples in the first and second with results for the first test only and results for all three tests with results for the first and second test. For strongyles, the Mini-FLOTAC method showed considerable variation, detecting 273 (out of 404) positive samples in the first count, 256 in the second and 249 in the third count. However, cumulative sensitivity only increased slightly owing to repeated counting, with 273 samples positive in the first, 277 in the first plus second and again 277 in the first plus second and third counts (Table 2). The same trends were observed for McMaster and semi-quantitative flotation: The number of positive samples decreased with each repetition and there was only a minimal (McMaster) or no (semi-quantitative flotation) cumulative sensitivity (Table 2). The McMaster method found 196 positives initially and 197 after the second and also after the third test round. The semi-quantitative flotation identified 213 positive samples in the first count and 200 and 194 positives after the second and all three tests, respectively. For *Moniezia* spp., both the McMaster and Mini-FLOTAC methods had identical results across all tests, detecting 9 and 31 positive samples, respectively. The semi-quantitative method varied slightly, with 17 positives initially and 18 after the second and third rounds of testing. Thus, the trend of decreasing sensitivity with increasing numbers of repetition (and time) was not observed.

**Table 2:** Sensitivity improvement from triple testing of each camel faecal sample (*n* = 404) for helminth eggs across the semi-quantitative flotation, McMaster and Mini-FLOTAC methods

Test procedure	Gastrointestinal nematodes			*Moniezia* spp.
	Strongyles	*Strongyloides* spp.	*Trichuris* spp.	
*Mini-FLOTAC*				
Number positive	277	14	1	31
First egg counting	273	10	0	31
Second egg counting	256	10	1	31
Third egg counting	249	5	0	31
First + second egg countings	277	14	1	31
All three egg countings	277	14	0	31
*McMaster*				
Number positive	197	14	3	9
First egg counting	196	13	3	9
Second egg counting	142	4	1	9
Third egg counting	132	3	0	9
First + second egg countings	197	14	3	9
All three egg countings	197	14	3	9
*Semi-quantitative flotation*				
Number positive	213	10	7	18
First egg counting	213	10	7	17
Second egg counting	200	7	5	12
Third egg counting	194	8	5	15
First + second egg countings	213	10	7	18
All three egg countings	213	10	7	18

### Comparison of coproscopic methods using inter-rater reliability analyses

The three methods were compared regarding agreement of samples identified as positive or negative with a gold standard defining a sample as positive if it was positive in any of the analyses methods (i.e. assuming a specificity of 100%) (Table 3). While Mini-FLOTAC displayed high sensitivity for strongyles, still 4.9% of positive strongyle samples were falsely diagnosed as negative on triplicate Mini-FLOTAC analyses. For the semi-quantitative flotation and McMaster methods, approximately 20.8% and 24.7% were false negative, respectively (Table 3). Cohen’s *κ* coefficients for strongyles using Mini-FLOTAC indicated almost perfect agreement (Cohen’s *κ* of 0.88), whereas moderate agreement was observed for the two other methods. A substantial agreement was observed for *Strongyloides* spp. with McMaster and Mini-FLOTAC, and moderate agreement with semi-quantitative flotation. The Cohen’s *κ* value of 0.82 for *Trichuris* spp. with the semi-quantitative flotation corresponded to almost perfect concordance and was much higher than for the other two methods. Once again, the Mini-FLOTAC method demonstrated the highest sensitivity for *Moniezia* spp., with Cohen’s *κ* values of 0.90 (Table 3).

**Table 3:** Cohen’s *κ* coefficients for the semi-quantitative flotation, McMaster and Mini-FLOTAC methods compared with the “gold standard” in 404 camel faecal samples investigated with each method in triplicate

Method	No. positive (%)	Sensitivity (%)	NPV (%)	Cohen’s *κ*
*Strongyles*				
Gold standard^a^	297 (73.5)	n.a	n.a	
Mini-FLOTAC	277 (68.6)	93.3	84.3	0.880
McMaster	197 (48.8)	66.3	51.7	0.511
SQ flotation	213 (52.7)	71.7	56.0	0.573
*Strongyloides* spp.				
Gold standard^a^	29 (7.2)	n.a	n.a	
Mini-FLOTAC	14 (3.5)	48.3	96.2	0.634
McMaster	14 (3.5)	48.3	96.2	0.634
SQ flotation	10 (2.5)	34.5	95.2	0.494
*Trichuris* spp.				
Gold standard^a^	10 (2.5)	n.a	n.a	
Mini-FLOTAC	1 (0.3)	10.0	97.8	0.178
McMaster	3 (0.7)	30.0	98.3	0.455
SQ flotation	7 (1.7)	70.0	99.2	0.820
*Moniezia* spp.				
*Gold standard*^a^	37 (9.2)	n.a	n.a	
Mini-FLOTAC	31 (7.7)	83.8	98.4	0.904
McMaster	9 (2.2)	24.3	92.9	0.369
SQ flotation	18 (4.5)	48.6	95.1	0.633

^a^The gold standard was defined by counting any sample that tested positive in at least one of the three replicates across any of the three methods: the semi-quantitative (SQ) flotation, McMaster and Mini-FLOTAC. This de facto sets the specificity to 100%, which might be a slight overestimate if contamination of laboratory equipment from previously analysed samples cannot be completely excluded in the field

*NPV* negative predictive value

### Comparison of the quantitative methods McMaster and Mini-FLOTAC in terms of faecal egg counts

**Fig. 3:**
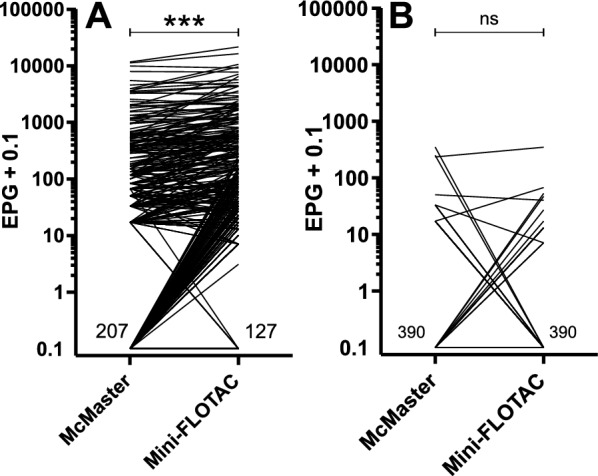
Comparison of the number of eggs per gram (EPG) of faeces obtained with the McMaster and Mini-FLOTAC methods for strongyle (**A**) and *Strongyloides* spp. (**B**) nematode eggs. To enable a logarithmic presentation, 0.1 was added to all values. For strongyle EPG, 127 and 207 samples tested negative with the Mini-FLOTAC and the McMaster method, respectively. The detected EPG were significantly higher for Mini-FLOTAC (mean 537.4, median 33, 75% percentile 271.5) than for McMaster (mean 330.1, median 0, 75% percentile 117.0) (*P* < 0.001, Wilcoxon matched-pairs signed rank test). For *Strongyloides* spp. EPG, the number of negative samples was equal between the two methods (390), and no significant differences were observed between McMaster and Mini-FLOTAC methods. ***P < 0.001

**Fig. 4:**
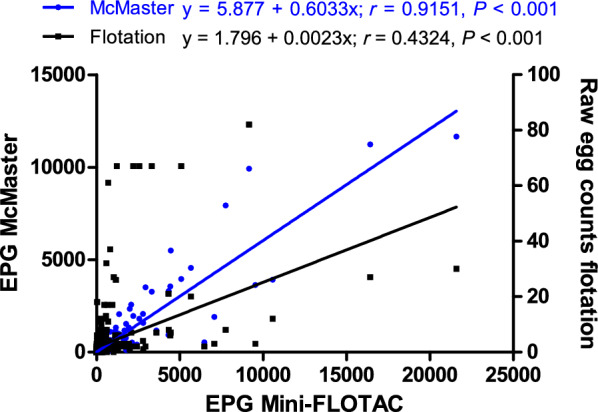
Pearson correlation between gastrointestinal strongyle eggs per gram (EPG) faeces obtained by Mini-FLOTAC method with EPG from the McMaster method and raw egg counts from the semi-quantitative flotation. The linear regression function is depicted as a continuous line. The formula for the regression functions, Pearson correlation coefficients and *P* values indicating a significant deviation of the slope from zero are displayed in the graphs

The Mini-FLOTAC method detected more positive samples than the McMaster method. Moreover, the mean EPGs for strongyle eggs were significantly higher when using Mini-FLOTAC, on the basis of the mean of three repeated counts (Fig. 3A). However, no significant difference was found between the two methods for detecting *Strongyloides* spp. (Fig. 3B). A linear regression and Pearson correlation analysis revealed a high correlation of EPG values between both methods for strongyle eggs (*r* = 0.915, *P* < 0.001). The slope of 0.60 shows that, overall, the EPG values for McMaster were only 60.0% of the EPGs for Mini-FLOTAC (Fig. 4). There was also a highly significant correlation between the EPG obtained using Mini-FLOTAC with the raw egg counts obtained by semi-quantitative flotation (*r* = 0.432, *P* < 0.001), but the correlation was much weaker than for Mini-FLOTAC and McMaster. While the Mini-FLOTAC EPG explains 84.0% of the variation observed in McMaster data, it explains only 19.0% of the variability in raw egg counts from semi-quantitative flotation. Considering that the EPG is calculated by multiplication of raw egg counts by 5, the slope of 0.0023 indicates that the raw egg count for semi-quantitative flotation was only 1.15% (0.0023 × 5 = 0.0115 = 1.15%) of that for Mini-FLOTAC.

### Comparison of quantitative methods (McMaster and Mini-FLOTAC) alongside the semi-quantitative flotation method for determining strongyle egg numbers

**Fig. 5:**
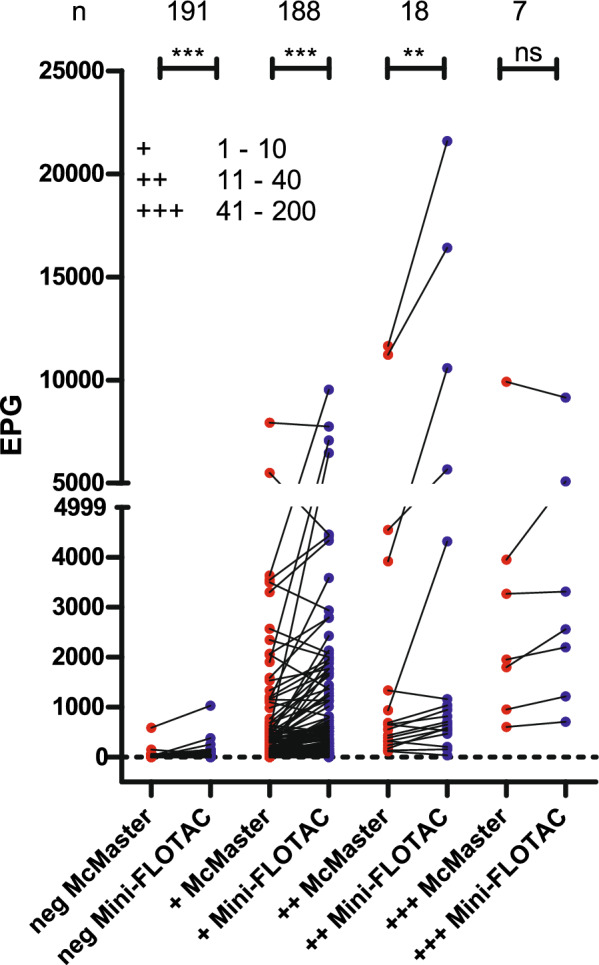
Comparison of strongyle faecal egg counts as eggs per gram (EPG) of faeces for each category from the semi-quantitative flotation method. Data were analysed using ANOVA followed by Tuckey’s post hoc test to compare McMaster and Mini-FLOTAC data within the same category from the semi-quantitative flotation method. Means are indicated by black horizontal lines. Categories in the semi-quantitative flotation method, ranging from negative, + (1–10 eggs), ++ (11–40 eggs) and +++ (41–200 eggs) were assigned according to the number of eggs counted (mean of triplicate count) under the microscope. The number of samples (*n*) in each category is provided at the top of the figure. *ns*, not significant; ***, **, *, *P* < 0.001, 0.01, 0.05, respectively

Samples were categorised according to the results of the mean (of triplicates) semi-quantitative flotation and then EPG values in each category between mean McMaster and mean Mini-FLOTAC (Fig. 5). One-way ANOVA and Spearman correlation analyses were conducted using the grouped egg data. For this dataset, very similar correlations of *ρ* = 0.697 and *ρ* = 0.708 (both *P* < 0.001) between the category from the semi-quantitative flotation and the EPGs from McMaster and Mini-FLOTAC methods were observed (Fig. 5). In Fig. 5, strongyle EPGs obtained by McMaster and Mini-FLOTAC are shown separately for each of the four grouped results. The mean (for all categories) and the median (for all categories except the category with samples negative by semi-quantitative flotation) were higher for the Mini-FLOTAC compared with the McMaster approach. Using a Wilcoxon matched-pairs signed rank test followed by *P* value correction, the EPGs were significantly higher for Mini-FLOTAC for the negative category and the categories + and ++ but not +++ (Fig. 5).

### Differences between McMaster and Mini-FLOTAC in terms of categorised outcomes

Identifying animals requiring treatment and distinguishing between different infection groups in camels would significantly enhance clinical diagnosis and treatment efficiency, thereby supporting the objectives of targeted selective treatment (TST) approaches. Henceforth, two different cut-off values were used: 200 and 500 strongyle EPG as determined by Mini-FLOTAC. For each cut-off value, samples were assigned into categories below (e.g. < 200), the same or above (e.g. ≥ 200) the threshold according to the Mini-FLOTAC data. Then, the McMaster strongyle EPG for each category was analysed (Table 4). The obtained results show that, irrespective of the cut-off value chosen, about 89.9–91.6% of the samples were placed into the same category by McMaster and Mini-FLOTAC, resulting in Cohen’s *κ* coefficients of 0.683 and 0.725, corresponding to substantial agreement.

**Table 4:** Inter-rater agreement between data obtained using McMaster and Mini-FLOTAC to categorise strongyle egg shedding intensities

Category Mini-FLOTAC^a^	Category McMaster^a^	Number^b^	% Category	% Correct % Incorrect	Cohen’s *κ*	Mean^c^ Median^c^	Range^c^
< 200	< 200	287	71.0	89.85 10.15	0.724	13.4 0	0–183
	≥ 200	2	0.5			375.0 375.0	317–433
≥ 200	< 200	39	9.7			100.0 117.0	0–183
	≥ 200	76	18.8			1643.0 641.5	200–11,650
Error rate 1 (%)^d^		39/115 (33.9)					
Error rate 2 (%)^e^		2/289 (0.7)					
< 500	< 500	324	80.2	91.58 8.42	0.683	44.6 0	0–493
	≥ 500	3	0.7			578.0 567.0	550–617
≥ 500	< 500	31	7.7			265.1 317.0	33–483
	≥ 500	46	11.4			2470.0 1333.0	500–11650
Error rate 1 (%)^d^		31/77 (40.3)					
Error rate 2 (%)^e^		3/327 (0.9)					

^a^McMaster and Mini-FLOTAC data were used to categorise samples using two different thresholds (200 and 500 eggs per gram (EPG) faeces)

^b^Number of samples out of *n* = 404 in total

^c^Mean, median and range of strongyle EPG determined by McMaster in the respective category

^d^Percentage of samples classified as above the threshold by Mini-FLOTAC but below the threshold by McMaster

^e^Percentage of samples classified as above the threshold by McMaster but below the threshold by Mini-FLOTAC

It is noteworthy that 39 (9.7%) of the total number of samples (*n* = 404) were categorised for strongyle EPGs equal to and higher than 200 using Mini-FLOTAC but below this threshold using McMaster (Table 4). Depending on the threshold, between 33.9% (39/115) and 40.3% (31/77) that were categorised above the threshold using Mini-FLOTAC were instead assigned to a category below the threshold by McMaster (error rate 1 in Table 4). There were only slight inconsistencies in egg counts between the two methods among samples with strongyle EPGs < 500 in Mini-FLOTAC, with McMaster reporting only three samples with EPG > 500 (range 550–617). Conversely, among samples assigned to a category above 500 EPG using Mini-FLOTAC, 31 samples were below the threshold with McMaster (EPG range 33–483). Among the samples assigned to a category above one of the thresholds using McMaster, 0.7% (cut-threshold 200 EPG) and 0.9% (threshold 500 EPG) were categorised into the opposite category by Mini-FLOTAC (error rate 2 in Table 4).

### Pooled faecal samples

**Fig. 6:**
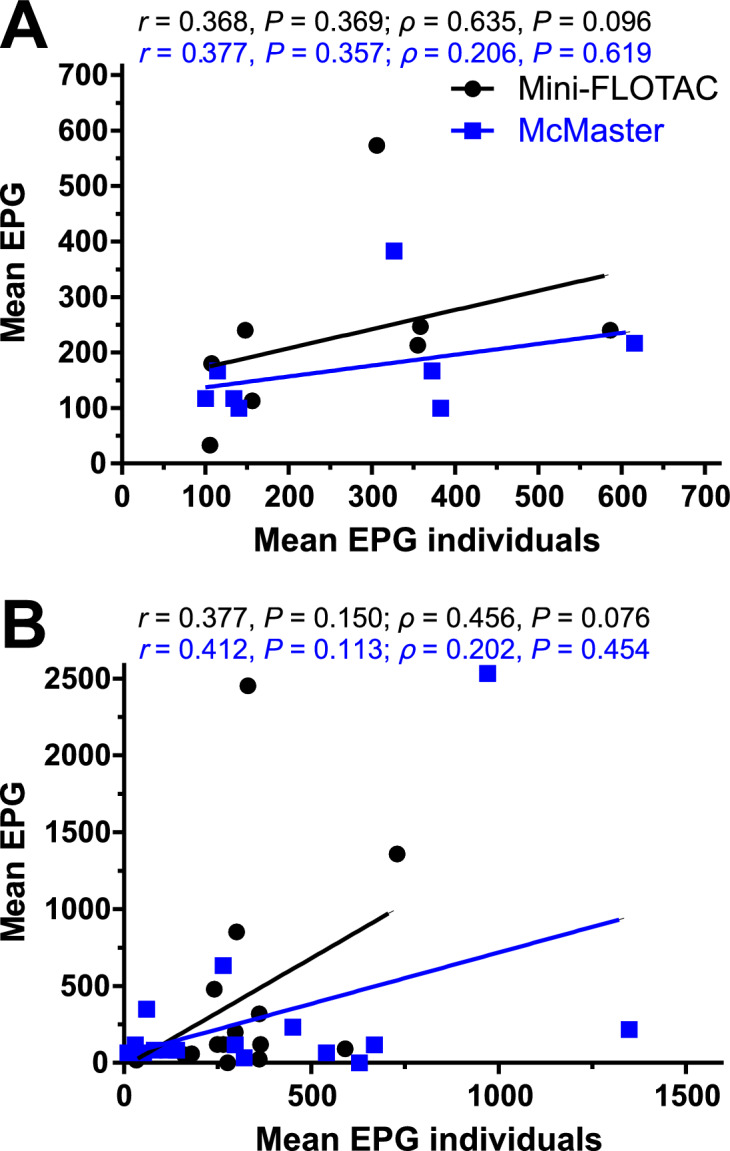
Correlation between strongyle faecal egg count (FEC) results from pooled samples (pool of ten (**A**) and pool of five (**B**)) and the mean strongyle FEC from individual samples in the pool for two different diagnostic methods (McMaster and Mini-FLOTAC). *r*, Pearson’s correlation coefficient; *ρ*, Spearman’s correlation coefficient

Figure 6 presents the correlation between strongyle FEC results from pooled samples and the mean strongyle FEC from individual samples in the pool. The analyses indicated that pooled FEC results generally did not show a positive correlation with the mean individual strongyle FEC, as reflected by Pearson correlation coefficients of *r* ≥ 0.368 (*P* ≥ 0.113). Results of Spearman correlation analyses also revealed no significant results. This lack of positive correlation was consistent across different pool sizes (five and ten) and with both the McMaster and Mini-FLOTAC methods (Fig. 6).

## Discussion

Faecal egg counting is crucial for diagnosing helminth infections in clinical settings, assessing treatments and detecting co-infections to guide therapy. The semi-quantitative flotation method is widely used for routine helminth diagnosis in humans and animals [3]. Although the McMaster method is a common quantitative technique, it has low sensitivity and precision [33]. Recently, Mini-FLOTAC has emerged as a promising alternative with improved sensitivity [21], but its effectiveness in various livestock species, including camels, is underexplored. Cost savings from pooled faecal sample analysis have been noted in small ruminants [10] and horses [34], but the representativeness of pooling for camels needs further investigation. This study aimed to identify optimal diagnostic tools by comparing the precision, sensitivity, inter-rater reliability and correlation of helminth egg counts in camels, including pooled samples.

Since the introduction of Mini-FLOTAC in 2013, several studies have compared it with the McMaster method for helminth egg counts, primarily focusing on strongyles. These comparisons have been conducted on bovines [35–40], sheep [33, 37, 41–44], goats [33, 44, 45] and llamas [37]. However, *Strongyloides* spp. counts have not been documented, and *Trichuris* spp. and *Moniezia* spp. counts have been compared in only a few ruminant species [40]. To the best of the authors’ knowledge, this study is the first to compare semi-quantitative flotation, McMaster and Mini-FLOTAC methods for detecting these four helminths in Old World camels.

The repeated measures using replicates drawn from the same or different tubes revealed only a few significant differences regarding EPGs, and these significant differences showed, in some cases, higher EPG estimates for the same and sometimes for different tubes, suggesting that effects of performing the homogenisation and sieving procedures separately or only once did not considerably impact on the results. Differences between SDs were also not meaningful, and the direction of differences was not consistent. For some samples, there were significant differences (A4, A5) in SD between samples, but for A4 there was a very high SD for McMaster from the same tube while for A5 both Mini-FLOTAC samples had very high SD values. In contrast, although there were no significant differences in CV (Fig. 2C), it was nevertheless obvious that the CV for the McMaster method was higher than for the Mini-FLOTAC method. One of the samples showed a very high CV using McMaster analyses performed from three different tubes but a CV of 0% for the McMaster analysis for the same analysis from the same tube. However, for the latter, the measured EPG was zero for a weakly positive sample (sample A6, mean EPG Mini-FLOTAC all repeats = 66.7). In contrast to the McMaster method, the Mini-FLOTAC method showed approximately constant CVs for all groups of analyses. With only five samples and a very high variation of McMaster for the weakly positive sample, the power was not high enough to confirm this effect statistically.

Mini-FLOTAC was the most sensitive of the three methods included and showed by far the best agreement with the gold standard, at least for strongyle and *Moniezia* eggs. Results for *Strongyloides* and *Trichuris* were not considered representative since the number of positive samples was low and the chosen flotation solution was also not optimal for *Trichuris* [46]. This enhanced sensitivity can be attributed to the larger volume and quantity of faecal samples examined by Mini-FLOTAC. Cringoli et al. [21] reported that the Mini-FLOTAC method enables analysis of a significantly larger faecal sample (up to 5 g) and directly examines 200 mg, in contrast to the McMaster method, which examines 20 mg in the protocol version used here (the maximum being 66.7 mg) [25], which corresponds to the differences in multiplication factors. Moreover, superior sensitivity of Mini-FLOTAC, particularly for low-density and thin-shelled helminth eggs such as strongyles, is likely due to its larger chamber size, which enhances egg flotation, and the 90° rotation of the reading disk before counting, which improves clarity and facilitates egg identification. Previous studies have shown Mini-FLOTAC’s superior precision and sensitivity over McMaster, Kato-Katz and FECPAK [33, 47, 48]. For example, Mini-FLOTAC, which uses a multiplication factor of 5, demonstrated better diagnostic performance in sheep and goats, with positive detection rates of 85.5% and 90.4%, respectively, compared with McMaster’s (multiplication factor of 50) 49.5% and 63.1% [33]. A study comparing Mini-FLOTAC (multiplication factor of 5) and McMaster (multiplication factor of 50) in cattle found that Mini-FLOTAC achieved 100% sensitivity across all GIN egg levels (10–500 EPG). In contrast, McMaster showed 100% sensitivity only for egg counts above 100 EPG, with sensitivity dropping to 0.0–66.6% for counts below this threshold, highlighting Mini-FLOTAC’s superior performance [38]. Similarly, Mini-FLOTAC (with a multiplication factor of 5 EPG) showed improved sensitivity in North American bison (80.6%) compared with McMaster (56.9%), which used a multiplication factor of 33.33 EPG [40]. In horses, Mini-FLOTAC (multiplication factor of 5) demonstrated superior sensitivity for strongyles but showed sensitivity similar to McMaster (multiplication factor of 50) for detecting *Anoplocephala* spp. and *Parascaris* spp. [49]. The superior sensitivity of Mini-FLOTAC (multiplication factor of 5) compared with McMaster (multiplication factor of 33) has also been demonstrated in detecting pig helminths, specifically strongyles and *Strongyloides ransomi* [50]. A study using egg-spiked chicken faecal samples (50–1250 EPG) found that Mini-FLOTAC (multiplication factor of 10) had 100% sensitivity compared with 97.1% for McMaster (multiplication factor of 50) [51]. One study compared the McMaster (multiplication factor of 33) and Mini-FLOTAC (multiplication factor of 5) methods for cattle, sheep, horses and llamas, a New World camelid [37]. The authors concluded that, owing to its high sensitivity, accuracy and precision, the Mini-FLOTAC is recommended for all four host species. In contrast to the method used to analyse the samples in the present study, the effect of repeating the same test twice or three times was minimal and not significant for all three methods. This is surprising since doing the test three times also decreases the multiplication factor by three, which should lead to higher sensitivity. This indicates that, in addition to the multiplication factor, representing the amount of faeces actually analysed under the microscope, additional method-inherent factors have a strong influence on sensitivity.

The 68.6% prevalence of strongyles in camels observed in the present study using the Mini-FLOTAC method is comparable to the 71.0% prevalence reported in Sudanese cattle by Mohammedsalih et al. [52] but lower than the 82.4% prevalence in Sudanese goats reported by Mohammedsalih et al. [2] in the same study area, using the same method and multiplication factor. Data for the same animal species using other faecal analysis methods are available in the database. The frequency of strongyles in camels (68.6%) in this study is higher than the 58.8% prevalence reported in dromedary camels in the Al-Butana area, River Nile State, Sudan, using the McMaster method [53]. This finding is also higher than the 21.8% prevalence reported in dromedary camels in Algeria using microscopic examination after the formalin–ether sedimentation and flotation method [54]. Similarly, a study in Kenya reported a 31.0% prevalence using the McMaster method [55], while a comparable prevalence of 64.0% was observed in the same animal species using the McMaster method in the central deserts of Iran [56]. Variations in prevalence across these studies may be due to differences in animal species, climate, husbandry practices and the number of animals included in each study.

In the present study, *Strongyloides* spp. eggs were found in 7.2% of the camel samples. Both the McMaster and Mini-FLOTAC methods showed similar sensitivities, demonstrating substantial agreement in detecting this nematode in camel faeces, while semi-quantitative flotation presented moderate agreement. A comparative study of at least two of these three methods for *Strongyloides* spp. is, to the knowledge of the authors, not available in the literature, and comparison of results is therefore impossible. The percentage of positive samples identified in camels in South Darfur State in the present study using the combined results of all three methods (gold standard) of 7.2% was slightly lower than the 9.0% reported for dromedary camels in Bangladesh using only the McMaster method [57] and the 11.2% reported in Algeria using microscopic examination after concentration by formalin–ether sedimentation, and flotation methods [54]. However, the frequency found in camels in the present study was higher than the 4.8% reported in Sudanese goats using Mini-FLOTAC in the same region [2].

The three methods evaluated in the present study successfully detected *Trichuris* spp. eggs in camel faeces, with an overall positive percentage of 2.5%. The semi-quantitative flotation method revealed better sensitivity, demonstrating a very high Cohen’s *κ* agreement with the gold standard. It detected more positive samples (7 out of 404) and showed the lowest rate of false negatives (30.0%), compared with false negative rates of 70.0% and 90.0% for the McMaster and Mini-FLOTAC methods, respectively. The low sensitivity of the McMaster and Mini-FLOTAC methods for detecting *Trichuris* spp. eggs was recently highlighted in North American bison faeces [40]. However, owing to the very small number of *Trichuris* spp. positive samples, any conclusions drawn from the *Trichuris* data should be considered only very carefully and the picture might change with larger data sets and in particular when flotation solutions with higher relative density (≥ 1.30).

*Moniezia* spp. eggs were detected in 9.2% of the camel samples examined in the present study. The Mini-FLOTAC method demonstrated almost perfect agreement (Cohen’s *κ* of 0.904) with the gold standard for detecting *Moniezia* spp. eggs in camel faeces, with the lowest false negative rate (16.2%) compared with the semi-quantitative flotation and McMaster methods, which showed fair to moderate agreement and higher false negative rates ranging from 51.4% to 75.7%, respectively. In contrast to the present study, Johnson et al. [40] found perfect agreement between Mini-FLOTAC and McMaster for detecting *Moniezia* spp. eggs. The differences between the two studies may be attributed to the use of different numbers of replicates, as they used the average count of eggs from three technical replicates for McMaster, with a multiplication factor of 33.33 EPG, whereas only one Mini-FLOTAC (multiplication factor of 5) was counted. The percentage of camels shedding *Moniezia* spp. eggs in the present study (9.2%) was higher than that reported by Bouragba et al. [54] in the same animal species in Algeria (4.3%), but a different faecal analysis method was used in Algeria. It was also similar to the prevalence reported in a study involving Bactrian and dromedary camels in the USA (7.8%), which used modified Wisconsin faecal flotation and McMaster methods [17]. Furthermore, the 9.2% positive percentage in the present study is higher than the prevalence of *Moniezia* spp. in Sudanese goats (5.9%) from the same region using Mini-FLOTAC [2]. Differences in the prevalence of *Moniezia* spp. across studies can be attributed to various factors, including the different faecal analysis methods used, variations between host animal species, the number of animals included in each study, the use of anticestodal drugs and climatic differences between study regions.

Repeating the helminth egg counting using the same method only slightly improved the overall sensitivity for detecting strongyles. This limited improvement can be attributed to the fact that the number of positive samples decreased across all methods as the number of replicates increased. Because these replicates were conducted sequentially, the duration that the eggs were exposed to the highly concentrated flotation solution (sodium chloride solution, relative density 1.20 [26]) also increased with each subsequent replicate. This extended exposure likely caused the strongyle eggs to deform, reducing the effectiveness of the method in later replicates. Interestingly, this effect was not observed for *Moniezia* spp. eggs, which likely have a more rigid and impermeable eggshell, preventing deformation. These findings indicate that simply tripling the number of replicates did not significantly enhance the detection of positive samples across any of the methods tested. This suggests that there are other limitations specific to each method that need further investigation. These limitations may stem from factors such as variations in sample preparation before flotation, the design of the flotation chamber and differences in visibility during sample analysis. For instance, the Mini-FLOTAC method offers a clearer view of the samples compared with the McMaster method, as most debris is removed from the optical field when the counting disc is rotated by 90° relative to the base of the apparatus. Another critical factor, particularly for detecting strongyles, is the flotation time. Prolonged exposure to the flotation solution is known to deform strongyle eggs [58], which likely contributed to the reduced sensitivity observed in later replicates in this study.

Refugia-based control strategies, such as TST, are beneficial in delaying the development of anthelmintic resistance. However, determining the optimal decision criteria for selecting individuals to treat in cases of subclinical infection remains a challenge. Faecal egg counts have been identified as one of the most reliable indicators for treatment decisions. Many previous TST studies recommend an EPG threshold of over 200 nematode eggs as the criterion for administering anthelmintic treatments, particularly in ruminants [30, 59, 60]. Therefore, sensitive faecal analysis methods are required for accurate FECs, particularly those with multiplication factors of ≤ 5 EPG [19]. In the present study, the Mini-FLOTAC method recovered more strongyle eggs in 68.6% of the samples, with fewer zero egg counts and a higher number of positive animals. Furthermore, 28.5% of animals had an EPG equal to or greater than the 200 EPG threshold for anthelmintic treatment. In comparison, the McMaster method detected strongyles with a 48.8% frequency, and only 19.3% of animals required anthelmintic treatment. This comparison supports the superiority of the Mini-FLOTAC method for FEC analysis and informing treatment decisions. The same is true if one applies a higher threshold of 500 EPG. Detailed clinical examinations together with FEC data and ideally information about co-infections will be required in the future to established a scientifically evaluated threshold for dromedaries.

Although pooling faecal samples can considerably reduce the workload in field sampling and laboratory analysis, our study found no significant agreement (*r* ≤ 0.412, *P* ≥ 0.113) between pooled and individual sample assessments for strongyle FEC. This disagrees with many previous studies on sheep [41, 42], cattle [61] and horses [34], which reported high correlations between individual and pooled FECs. The differences might be due to differences in animal species and age composition [10, 34], helminth egg distribution in the faecal matrix [62], faecal consistency [63] and the influence of the tropical savannah climate [62]. Effects of the animal species have been described previously. In particular, correlation and agreement between pooled and individual FECs have been shown to be better in sheep than in goats [10]. The climate potentially also has a notable effect [62]. Most prior studies on pooled faecal samples were conducted in temperate climates, differing significantly from the conditions in the present study, which may have an effect on faecal consistency. In fact, the faecal samples collected from camels in Sudan were very dry and hard, and this might have effects on the efficacy of sample homogenisation during pooling. These data show that further analyses are required to evaluate factors that might limit the suitability of pooled samples as cost-effective for analysing samples of individual animals.

Although the time required to conduct the faecal analysis of the samples using the two quantitative methods was not recorded in the present study, previous studies comparing the McMaster and Mini-FLOTAC methods have established that the Mini-FLOTAC procedure requires significantly longer hands-on time (13 min per sample) compared with the McMaster method (7 min per sample) [48, 51, 64].

## Conclusions

In this study, both semi-quantitative flotation and two quantitative methods, i.e. McMaster and Mini-FLOTAC, effectively identified four helminth genera/groups, including strongyles, *Strongyloides* spp., *Trichuris* spp. and *Moniezia* spp., in the examined camels. The Mini-FLOTAC method emerged as a viable alternative to the McMaster method for quantifying strongyle eggs, *Strongyloides* spp., and *Moniezia* spp. Notably, the Mini-FLOTAC consistently outperformed both the semi-quantitative flotation and the McMaster method, recovering a significantly higher number of gastrointestinal helminths from naturally infected camel faeces, particularly for strongyles, *Strongyloides* spp. and *Moniezia* spp. Owing to the much higher raw egg counts (higher EPG and smaller multiplication factor), the Mini-FLOTAC method is also the method of choice to conduct FECRTs with high statistical power in camels and to guide treatment decisions in camels.

## Supplementary Information

Additional file 1.

## Data Availability

The relevant information has been included in the manuscript. Data analysed for this manuscript are available from the corresponding author upon request.

## Abbreviations

CI: Confidence interval
EPG: Eggs per gram
FECR: Faecal egg count reduction
FECRT: Faecal egg count reduction test
GINs: Gastrointestinal nematodes
TST: Targeted selective treatment
