# Supplementary material for: Comparative assessment of Mini-FLOTAC, McMaster and semi-quantitative flotation for helminth egg examination in camel faeces
Source: Parasit Vectors. 2025 Jan 12;18:5. doi: 10.1186/s13071-024-06637-3 (PMC11726973; doi:10.1186/s13071-024-06637-3)
Supplement: Supplementary file 1 — Additional file 1. [file 13071_2024_6637_MOESM1_ESM.pdf]

A

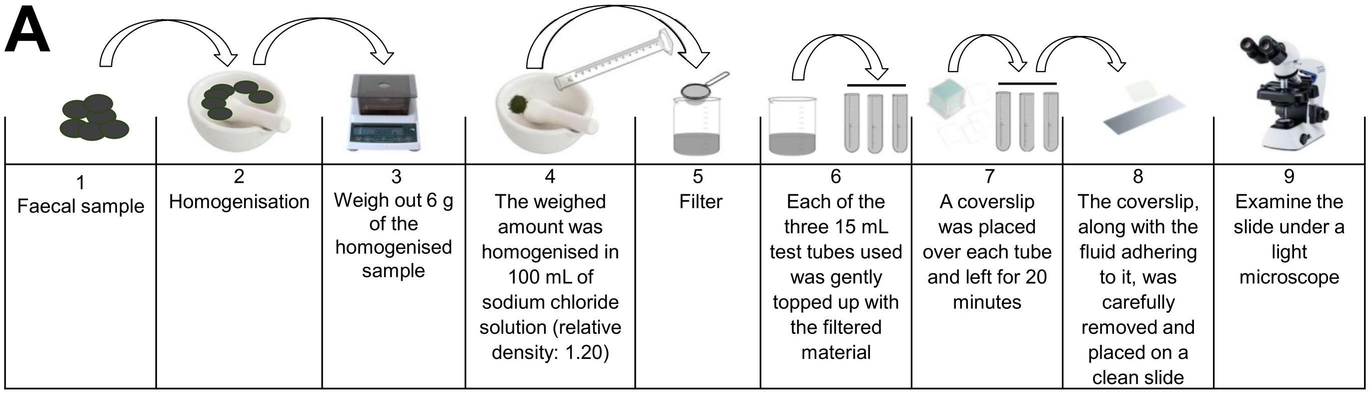

1	2	3	4	5	6	7	8	9
Faecal sample	Homogenisation	Weigh out 6 g of the homogenised sample	The weighed amount was homogenised in 100 mL of sodium chloride solution (relative density: 1.20)	Filter	Each of the three 15 mL test tubes used was gently topped up with the filtered material	A coverslip was placed over each tube and left for 20 minutes	The coverslip, along with the fluid adhering to it, was carefully removed and placed on a clean slide	Examine the slide under a light microscope

B

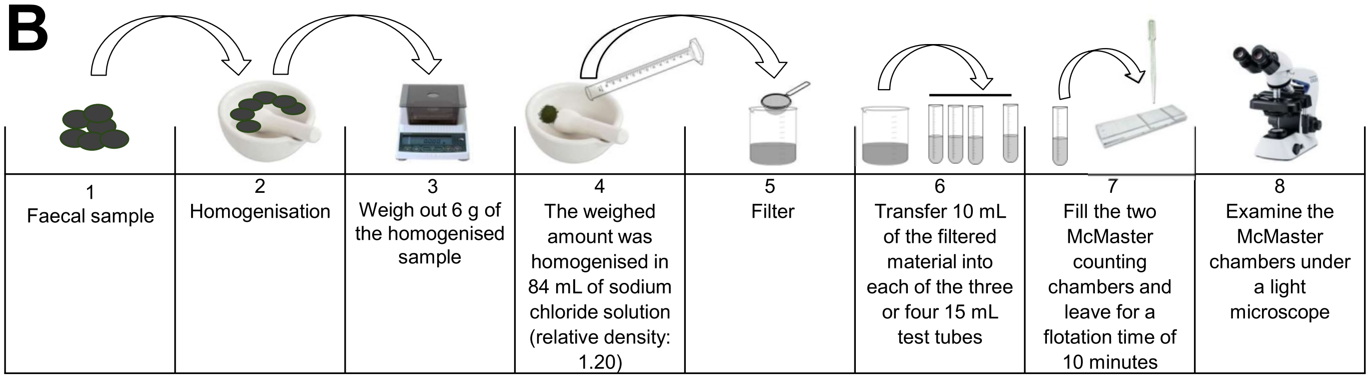

1	2	3	4	5	6	7	8
Faecal sample	Homogenisation	Weigh out 6 g of the homogenised sample	The weighed amount was homogenised in 84 mL of sodium chloride solution (relative density: 1.20)	Filter	Transfer 10 mL of the filtered material into each of the three or four 15 mL test tubes	Fill the two McMaster counting chambers and leave for a flotation time of 10 minutes	Examine the McMaster chambers under a light microscope

C

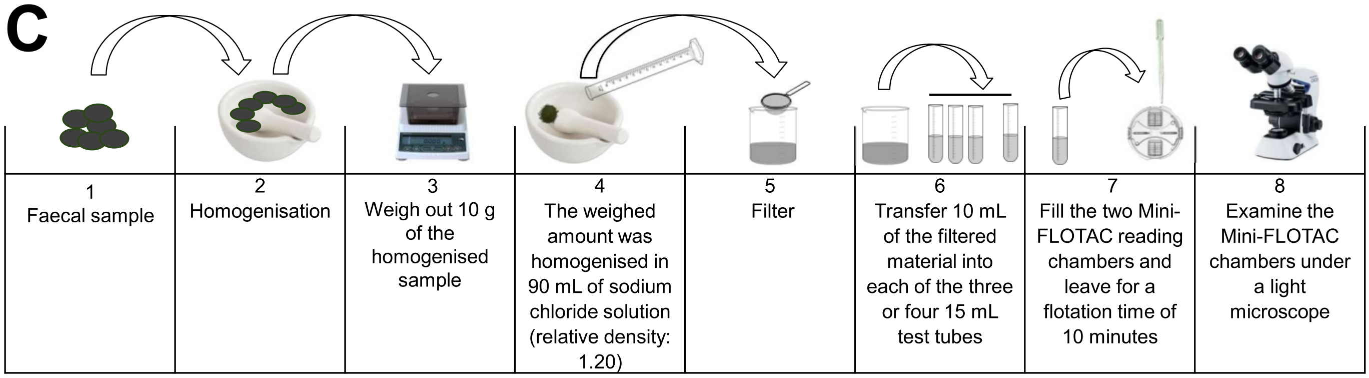

1	2	3	4	5	6	7	8
Faecal sample	Homogenisation	Weigh out 10 g of the homogenised sample	The weighed amount was homogenised in 90 mL of sodium chloride solution (relative density: 1.20)	Filter	Transfer 10 mL of the filtered material into each of the three or four 15 mL test tubes	Fill the two Mini-FLOTAC reading chambers and leave for a flotation time of 10 minutes	Examine the Mini-FLOTAC chambers under a light microscope

Additional file 1: Fig. S1 The operating steps of the semi-quantitative flotation, McMaster, and Mini-FLOTAC methods. (A) The nine operating steps of the semi-quantitative flotation method. (B) The eight operating steps of the McMaster method. (C) The eight operating steps of the Mini-FLOTAC method. Faecal samples were collected from the rectum of individual camels (*n*=410) and homogenised, individually, with a pestle and mortar. Sample weights were measured using a 0.001 g sensitivity balance (Shimadzu BL220H): 6 g for the semi-quantitative flotation and McMaster methods, and 10 g for Mini-FLOTAC. The samples were mixed with saturated sodium chloride solution (relative density: 1.2): 84 mL for McMaster, 90 mL for Mini-FLOTAC, and 100 mL for semi-quantitative flotation. The mixtures were filtered through a 0.3 mm mesh tea strainer into a 400 mL measuring beaker. For semi-quantitative flotation, the suspension was divided into three 15 mL test tubes for triplicate counting. Each tube was topped up to form a convex meniscus, covered with a coverslip, and left for 20 minutes. The coverslip was then transferred to a slide for examination under a light microscope (Olympus CX23) at 100× magnification. For McMaster and Mini-FLOTAC, the filtered suspension was divided into three or four 10 mL aliquots (depending on the experiment) in 15 mL test tubes. The two McMaster chambers were filled with 0.15 mL each, and the two Mini-FLOTAC chambers with 1 mL each. In both methods, eggs floated for 10 minutes before microscope examination at 100× magnification. Helminth eggs per gram of faeces were calculated by multiplying the observed egg count by 50 for McMaster and by 5 for Mini-FLOTAC.
